# Poly-*S*-Nitrosated Albumin as a Safe and Effective Multifunctional Antitumor Agent: Characterization, Biochemistry and Possible Future Therapeutic Applications

**DOI:** 10.1155/2013/353892

**Published:** 2013-12-30

**Authors:** Yu Ishima, Ulrich Kragh-Hansen, Toru Maruyama, Masaki Otagiri

**Affiliations:** ^1^Department of Biopharmaceutics, Graduate School of Pharmaceutical Sciences, Kumamoto University, 5-1 Oe-honmachi, Kumamoto 862-0973, Japan; ^2^Center for Clinical Pharmaceutical Sciences, Kumamoto University, 5-1 Oe-honmachi, Kumamoto 862-0973, Japan; ^3^Department of Biomedicine, University of Aarhus, 8000 Aarhus C, Denmark; ^4^Department of Biopharmaceutics, Faculty of Pharmaceutical Sciences, Sojo University, 4-22-1, Ikeda, Kumamoto 860-0082, Japan; ^5^DDS Research Institute, Sojo University, 4-22-1 Ikeda, Kumamoto 860-0082, Japan

## Abstract

Nitric oxide (NO) is a ubiquitous molecule involved in multiple cellular functions. Inappropriate production of NO may lead to disease states. To date, pharmacologically active compounds that release NO within the body, such as organic nitrates, have been used as therapeutic agents, but their efficacy is significantly limited by unwanted side effects. Therefore, novel NO donors with better pharmacological and pharmacokinetic properties are highly desirable. The *S*-nitrosothiol fraction in plasma is largely composed of endogenous *S*-nitrosated human serum albumin (Mono-SNO-HSA), and that is why we are testing whether this albumin form can be therapeutically useful. Recently, we developed SNO-HSA analogs such as SNO-HSA with many conjugated SNO groups (Poly-SNO-HSA) which were prepared using chemical modification. Unexpectedly, we found striking inverse effects between Poly-SNO-HSA and Mono-SNO-HSA. Despite the fact that Mono-SNO-HSA inhibits apoptosis, Poly-SNO-HSA possesses very strong proapoptotic effects against tumor cells. Furthermore, Poly-SNO-HSA can reduce or perhaps completely eliminate the multidrug resistance often developed by cancer cells. In this review, we forward the possibility that Poly-SNO-HSA can be used as a safe and effective multifunctional antitumor agent.

## 1. Nitric Oxide Delivery Systems

Nitric oxide (NO) is a unique, diffusible molecular messenger that plays a central role in mammalian physiology and pathophysiology [1–7]. The effects of NO are pleiotropic, including vascular smooth muscle relaxation [8, 9], inhibition of platelet aggregation [10], and regulation of immune and neuronal functions [11]. However, under certain circumstances NO can be cytotoxic. For example, high concentrations of NO can inhibit tumor cell growth and induce apoptosis. Recent studies have revealed that NO is associated with not only apoptosis of cancer cells but also with inhibition of cancer progression and metastasis, as well as cancer angiogenesis. It also functions as a modulator for chemo/radio/immunotherapy. Unfortunately, despite such highly useful properties, the use of NO has been impeded by the fact that its *in vivo* half-life is so short (~0.1 s) that NO itself cannot be used as a therapeutic agent. Therefore, pharmacologically active compounds that can release NO or lead to its formation in the body have been synthesized. For example, organic nitrates and nitrite esters have been used for many years to treat patients with ischemic heart disease. However, there are well-known side effects and limitations to these NO donors, including potentially adverse hemodynamic effects, drug tolerance, lack of selectivity and limited bioavailability [12, 13]. Thus, it is essential to develop reliable NO donors with better pharmacological and pharmacokinetic parameters.

It is also important to be able to control reaction selectivity and dose of the NO donor against reactive oxygen species such as in NO therapy in inflammatory diseases. A high concentration of NO produced by inducible nitric oxide synthase (iNOS) is protective against bacterial infection in inflammatory processes, but too much NO will induce apoptosis and cellular damage [1, 15–17]. The latter effect is due to the formation of peroxynitrite (ONOO^−^), the reaction product of the interaction between superoxide (O_2_^−^) and NO, a potent proinflammatory nitroxide implicated in acute and chronic inflammatory conditions of many etiologies [18–21]. In addition, tissue injury and inflammation often accompany rapid development of hypersensitivity to noxious and nonnoxious stimuli (hyperalgesia and allodynia, resp.). In fact, sodium nitroprusside, which has been parenterally administered for the treatment of hypertension and heart failure, also induces an increase in the vascular production of superoxide leading to the formation of ONOO^−^, which is associated with cytotoxic effects of sodium nitroprusside [22, 23].

Local application of NO may be a very effective and safe form of NO therapy. To develop a method for the targeted delivery of NO, several groups of researchers have synthesized NO donors that hopefully can release NO selectively at a target site. For example, O(2)-vinyl-1-(pyrrolidin-1-yl)diazen-1-ium-1,2-diolate (V-PYRRO/NO) and 2-(acetyloxy)benzoic acid-3-(nitrooxymethyl)phenyl ester (NCX-1000) can selectively release NO in the liver. NO release from V-PYRRO/NO is mediated by cytochrome P450 which removes the vinyl group of the drug to generate free PYRRO/NO ion [24–26]. NCX-1000 is a prototype of a family of NO-releasing derivatives of ursodeoxycholic acid. The two compounds are selectively metabolized in the liver and biologically active NO enters the liver microcirculation without a detectable effect on systemic circulation [27]. However, these NO donors have not yet been applied to clinical situations, because their reaction mechanisms are not yet fully clarified. Thus, although NO release from V-PYRRO/NO is mediated by cytochrome P450, the isoform of cytochrome P450 catalyzing the process has not been identified. The enzyme that mediates NO release from NCX-1000 is still unknown. The enzymes that mediate NO release from V-PYRRO/NO and NCX-1000 must be identified in order to optimize their therapeutic efficacy [28].

In our search for a reliable and safe NO donor, we have followed a different approach, namely, to examine the possibility of using a NO-traffic protein. By a NO-traffic protein is meant a protein with (i) high efficiency of *S*-nitrosation, (ii) high stability of the *S*-nitroso form in the circulation, and (iii) high efficiency of *S*-transnitrosation into cells which need NO. As a candidate in this respect we focus on human serum albumin (HSA), because HSA is the most abundant plasma protein (35–50 g/L) and because endogenous *S*-nitrosothiol in human plasma is largely associated with HSA [31, 32].

## 2. Native, *S*-Nitrosated HSA as a NO Carrier

*S*-nitrosated HSA (SNO-HSA) is significantly more stable than low molecular weight *S*-nitrosothiols [31, 32]. In addition to us, also other researchers have attempted to produce NO delivery systems using a NO-albumin conjugate. Thus, Marks et al. [34] and Ewing et al. [35] have synthesized a macromolecular *S*-nitrosothiol, Poly-SNO-BSA, in which several *S*-nitrosothiols are formed in bovine serum albumin (BSA) after reduction of the protein's disulfide bonds. Independently, Beak et al. have developed a macromolecular NONOate, diazeniumdiolated BSA, in which several NONOate moieties have been conjugated to native BSA [36]. In a porcine coronary angioplasty model, the two BSA-forms, Poly-SNO-BSA and diazeniumdiolated BSA, were applied locally to a site of vascular injury and showed high retention at the administration site and reduced platelet attachment and activation. These effects were due to high binding of the modified albumins to the injured vessel. In the development of targeted NO delivery systems for intravenous use, tissue distribution characteristics of the NO-carrier conjugate should be evaluated *in vivo* in order to identify the various obstacles to targeted delivery, such as extensive uptake by mononuclear phagocyte systems and rapid loss by glomerular filtration. Katsumi et al. have examined the pharmacokinetic properties of SNO-BSA. The results showed that serum albumin is a promising carrier to control pharmacokinetic properties of NO after intravenous injection, because *S*-nitrosated albumin shows a relatively high retention in the blood circulation after intravenous injection into mice. However, targeted NO delivery after intravenous injection using a macromolecular carrier has not been successfully achieved so far [37].

To achieve targeted NO delivery from SNO-HSA after intravenous injection, we need to understand the method of *S*-nitrosation, the structure of HSA, and its biological fate in detail. Therefore, we have recently examined the structure and the biological effects of Mono-SNO-HSA, HSA with one (or less) *S*-nitrosothiol, and Poly-SNO-HSA.

**Figure 1:**
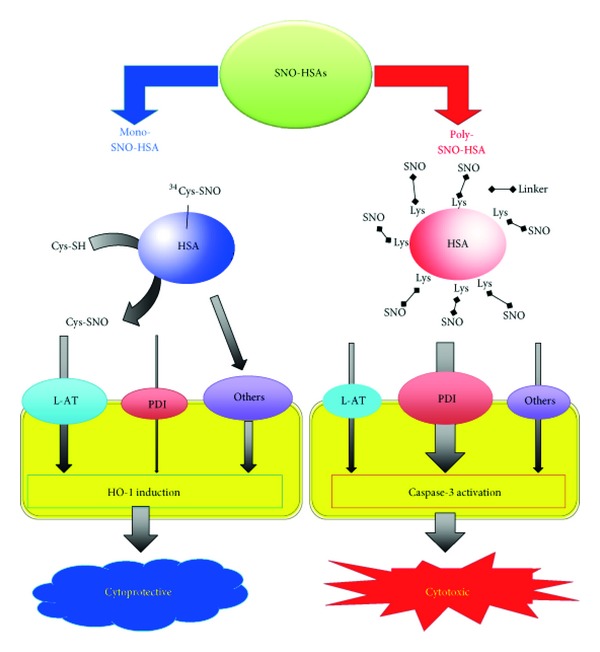
Differences in the mechanisms and consequences of NO traffic from Mono-SNO-HSA and Poly-SNO-HSA to cells. NO transfer from the SNO group of Cys-34 on Mono-SNO-HSA to the cell is partly mediated by the L-amino acid transporter (L-AT) via *S*-transnitrosation to free low molecular weight thiol. By contrast, NO transfer from Poly-SNO-HSA is mainly mediated by cell-surface protein disulfide isomerase (PDI) without *S*-transnitrosation to free low molecular weight thiol. The relatively slow transfer of NO from Mono-SNO-HSA avoids the presence of high intracellular NO concentrations and leads to cytoprotective activity through heme oxygenase-1 (HO-1) induction. On the other hand, the NO influx from Poly-SNO-HSA is very fast and pronounced and leads to cell death caused by apoptosis [14].

Endogenous *S*-nitrosated human serum albumin (Mono-SNO-HSA) is a large molecular weight NO carrier in human plasma, which has shown many beneficial effects in different animal models. In an attempt to construct more efficient SNO-HSA preparations, we have prepared SNO-HSA with many conjugated SNO groups (Poly-SNO-HSA) using chemical modification with 2-iminothiolane. We have compared the properties of such a preparation to those of Mono-SNO-HSA using C26 and HepG2 cells [14]. We found that cellular uptake of NO from Mono-SNO-HSA partly takes place via low molecular weight thiol, and it results in cytoprotective effects by induction of heme oxygenase-1. By contrast, transfer of NO from Poly-SNO-HSA into the cells was faster and more pronounced. The influx mainly takes place by cell-surface protein disulfide isomerase. Instead of cytoprotection, the considerable NO inflow resulted in apoptotic cell death caused by reactive oxygen species (ROS) induction, caspase-3 activation, and other means. Thus, increasing the number of SNO groups on HSA does not simply intensify the cellular responses to NO but can also result in very different effects (Figure 1). The number of moles of NO per mole of HSA in the Poly-SNO-HSA preparation used in our studies was estimated to be 6.6 ± 0.5 mol NO/mol HSA. The half-life (T_1/2_) of Poly-SNO-HSA in phosphate buffered saline, pH 7.4, is 21 ± 3 days at 25°C. On the other hand, T_1/2_ of Poly-SNO-HSA in mice is only 2.1 ± 0.3 h. A metabolite of Poly-SNO-HSA, iminothiolane-modified HSA which had been exposed to UV-light, had no modified effect on LDH release as compared with normal HSA [14]. Thus, Mono-SNO-HSA could be a cytoprotective agent, whereas Poly-SNO-HSA could have potential as an antitumor agent. In this review, we discuss the possibility of using Poly-SNO-HSA as a safe and effective multifunctional antitumor agent in biological systems.

## 3. Nitric Oxide Donor as Cancer Therapeutics Agent

NO is a cell signaling molecule that can be a potent inducer of cell death in cancers at elevated concentrations [38]. For example, increased intracellular NO levels lead to growth inhibition of both androgen-dependent and castration-resistant prostate tumors through a mechanism that involves androgen receptor function inactivation by *S*-nitrosylation of a single C601 residue present in the DNA-binding domain [39]. Furthermore, NO donors such as spermine/NO and diethylenetriamine/NO present cytotoxic activity on ovarian cancer cell lines, mainly through induction of apoptosis through inhibition of phosphorylation of STAT3 and AKT3 signaling proteins [40]. NO-donating nonsteroidal anti-inflammatory drugs (NSAIDs), especially NO-aspirin (NO-ASA), have also been shown to be able to reduce the growth of cultured HT-29 colon adenocarcinoma cells [41]. NO-ASA consists of traditional ASA to which an NO-releasing moiety is bound via a spacer. This agent induces oxidative stress by increasing intracellular peroxide and superoxide, thereby inducing apoptosis via activation of the intrinsic apoptosis pathway [42]. More recently, glutathione-*S*-transferase activated NO generators have shown some promise for NO therapy of cancer [43]. Intravenous injection of one such agent, JS-K, into mice bearing subcutaneously implanted multiple myeloma tumors on the flank, resulted in an impressive inhibition of tumor growth and induction of extensive apoptosis throughout the tumor. Finally, Duan et al. have designed a polymeric carrier system to deliver nitric oxide locoregionally to tumorigenic tissues at micromolar concentrations, and treatment of tumor-bearing nude mice with this polymeric carrier resulted in 50% tumor inhibition and in a 7-week extension of the average survival time, compared to intravenous therapy with the above mentioned prodrug JS-K [44]. Thus, cancer therapy by NO donors has been actively investigated. However, free NO can also be toxic to normal tissues, and chronic exposure at low levels can induce tumor growth. In addition, systemic use of NO donor drugs at high doses can result in hypotension [40]. In comparison, HSA has several advantages as a tumor targeting NO carrier as mentioned in Native, *S*-Nitrosated HSA as a NO Carrier.

## 4. Direct Antitumor Effect of Poly-SNO-HSA via Apoptosis

Poly-SNO-HSA as a NO donor has been investigated for its potential therapeutic applications, but there had been no reports describing the effects of Poly-SNO-HSA on cancer. Therefore, our research group produced Poly-SNO-HSA using the chemical linker 2-iminothiolane and observed that this HSA form can induce apoptosis in cancer cells [14]. The apoptosis occurs via activation of the intrinsic apoptosis pathway in, for example, murine colon 26 carcinoma cells and in the rat tumor cell line LY-80 (a variant of Yoshida sarcoma) both *in vivo* and *in vitro*.

**Figure 2:**
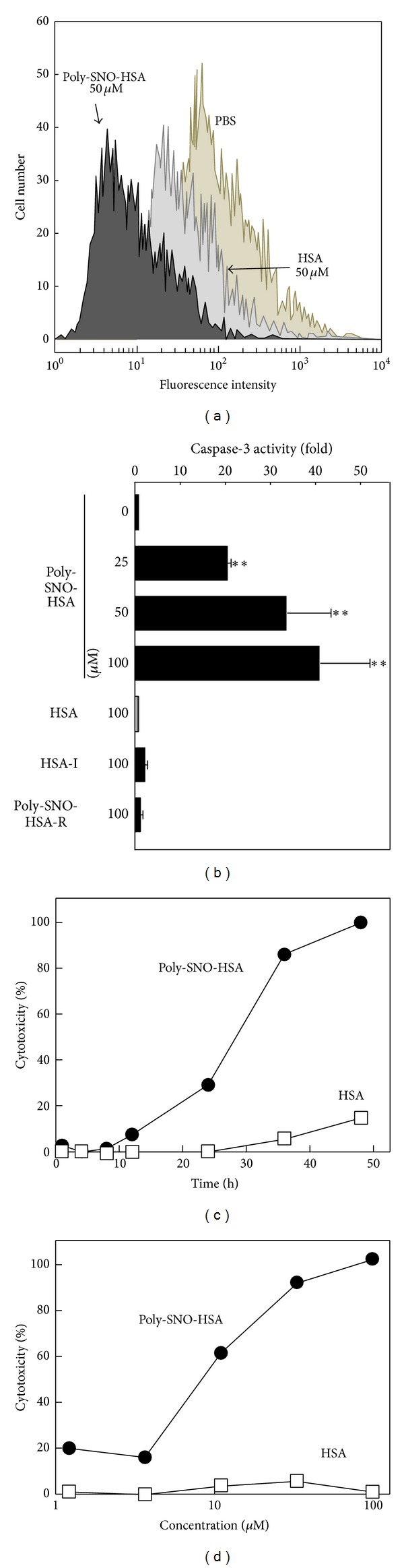
(a) Mitochondrial transmembrane potential is altered by Poly-SNO-HSA treatment. LY-80 cells were cultured with either phosphate buffered saline (PBS), 50 *μ*M HSA, or 50 *μ*M Poly-SNO-HSA for 2 h, followed by addition of rhodamine 123. Results are for one representative experiment. (b) Activation of caspase-3 after Poly-SNO-HSA treatment. LY-80 cells were incubated with either PBS (0), 100 *μ*M of HSA, HSA-I or Poly-SNO-HSA-R or with different concentrations of Poly-SNO-HSA for 24 h. HSA-I and Poly-SNO-HSA-R represent 2-iminothiolane modified HSA and UV-reduced Poly-SNO-HSA, respectively. Results are means ± SD of three separate experiments. (c) Effect of Poly-SNO-HSA on LDH release (cytotoxicity). LY-80 cells were incubated for the indicated times with 100 *μ*M HSA (open squares) or 100 *μ*M Poly-SNO-HSA (closed circles). (d) Effect of Poly-SNO-HSA on LDH release (cytotoxicity). LY-80 cells were treated for 48 h with various concentrations of HSA (open squares) or Poly-SNO-HSA (closed circles). Results from three separate experiments are presented as means. **P < 0.01, compared with control.

The process was studied in some detail. For example, mitochondria seem to play a pivotal role in the regulation of this process in mammals, because it is believed that loss of mitochondrial membrane potential is an essential element of apoptosis. To evaluate the effects of Poly-SNO-HSA on mitochondrial function and membrane potential, LY-80 cells were loaded with a mitochondrion-selective fluorescent cation (rhodamine 123), and we found that Poly-SNO-HSA treatment decreased rhodamine fluorescent intensity in a dose-dependent manner [29, 30]. HSA also attenuated fluorescence, but to a lesser extent than Poly-SNO-HSA (Figure 2(a)). These observations indicate that Poly-SNO-HSA induces depolarization of the mitochondrial membrane.

Caspase-3 is a cell-death protease that is involved in the downstream execution phase of apoptosis, during which cells undergo morphological changes, such as DNA fragmentation, chromatin condensation, and formation of apoptotic bodies. As compared to controls, LY-80 cells treated with 25, 50, or 100 *μ*M Poly-SNO-HSA showed relative increases in caspase-3 activity of 21-, 34-, and 42-fold, respectively (Figure 2(b)). The caspase-3 activity of HSA-treated cells was equivalent to that of cells treated with buffer alone. As additional controls, in order to elucidate the effect of 2-iminothiolane on the activation of caspase-3, HSA-2-iminothiolane modified (HSA-I) and UV-reduced Poly-SNO-HSA (Poly-SNO-HSA-R) were also incubated with LY-80 cells. Predictably, Poly-SNO-HSA-R as well as HSA-I did not activate caspase-3, which suggested that 2-iminothiolane did not participate in the activation of caspase-3 and that NO bound via functional thiols introduced by reaction of 2-iminothiolane with amino groups of lysine residues on HSA might be more efficient for its release than NO bound to free thiols obtained by reduction (Figure 2(b)). As an interesting observation supporting this result, also uptake of NO from Poly-SNO-HSA to HepG2 cells was much higher than that from Mono-SNO-HSA [14]. To further confirm that Poly-SNO-HSA induced apoptosis in LY-80 cells, DNA fragmentation, which is a morphological change characteristic of the execution phase of apoptosis, was examined [30]. DNA fragmentation was observed in LY-80 cells after 4 h of incubation with 100 *μ*M Poly-SNO-HSA and stabilized after 8 h. By contrast, DNA fragmentation was not detected until 24 h of incubation with 100 *μ*M HSA. Moreover, the DNA ladder observed after 12 h of incubation in Poly-SNO-HSA increased in a dose-dependent manner. To determine the mechanism by which Poly-SNO-HSA causes DNA fragmentation, LY-80 cells were simultaneously incubated with Poly-SNO-HSAs and Z-VAD-FMK (a caspase inhibitor). DNA fragmentation induced by 100 *μ*M Poly-SNO-HSA was completely abolished by treatment with Z-VAD-FMK, indicating that caspases are positive, upstream regulators of the DNA fragmentation that is elicited by Poly-SNO-HSA.

To determine the effect of Poly-SNO-HSA on cell growth, the viability of LY-80 cells was examined after treatment with either HSA or various concentrations of Poly-SNO-HSA. We found that Poly-SNO-HSA inhibited growth of LY-80 cells in a concentration-dependent manner. HSA also tended to abrogate cell proliferation, but to a lesser extent than Poly-SNO-HSA. To further characterize Poly-SNO-HSA-induced LY-80 cell death, cytotoxicity was examined using an assay of lactate dehydrogenase (LDH) activity (Figure 2(c)). LDH is a stable enzyme that is rapidly released from cells into the cell culture medium upon damage to the plasma membrane. Cell death increased with incubation time for the cultures incubated with 100 *μ*M Poly-SNO-HSA. After 48 h of incubation, LDH was released from nearly all cells. In contrast, HSA was not cytotoxic. We also observed that the cytotoxicity of Poly-SNO-HSA towards LY-80 cells is dose-dependent (Figure 2(d)). These, and the above, results suggest that Poly-SNO-HSA induces cell death via activation of an intrinsic apoptosis-signaling pathway.

**Figure 3:**
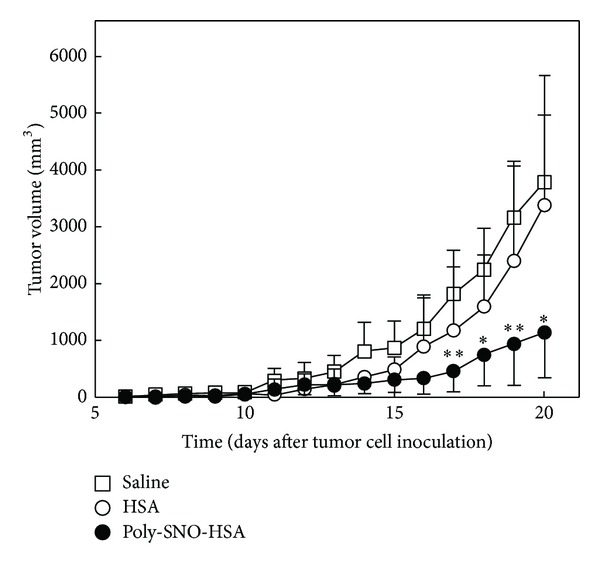
Effect of Poly-SNO-HSA on tumor growth in LY-80 tumor-bearing rats. LY-80 tumor-bearing rats were given daily intratumor injections of saline (5 mL/kg; open squares), HSA (10 *μ*mol/5 mL/kg; open circles), or Poly-SNO-HSA (10 *μ*mol/5 mL/kg; closed circles) for 7 days (day 5–11 after inoculation with tumor cells). Results are means ± SD; *n* = 4 animals per experimental group. *Statistically significant reduction in tumor growth as compared with treatment with saline (*P* < 0.05) or HSA (*P* < 0.05) at the corresponding time. **Statistically significant reduction as compared with treatment with saline (*P* < 0.01) or HSA (*P* < 0.05) at the corresponding time [29, 30].

To investigate the antitumor effects of Poly-SNO-HSA *in vivo*, LY-80 tumor-bearing rats received either intravenous or direct intratumor injections of either saline, HSA, or Poly-SNO-HSA. Mean tumor volume increased with time in the saline-treated group. A similar trend was observed in the HSA-treated group. However, tumor growth in animals that received direct intratumor injections of Poly-SNO-HSA was only one-third that observed in the saline- and HSA-treated animals (Figure 3). This observation suggests that Poly-SNO-HSA has antitumor effects *in vivo*, presumably due to induction of apoptosis. We observed similar results, when we used mice bearing other cell types such as murine colon 26 carcinoma cells or SW480 cells [29].

The latter study [29] also revealed that treatment with Poly-SNO-HSA caused no significant changes in total serum protein, serum creatinine, blood urea nitrogen, aspartate aminotransferase, or alanine aminotransferase. These findings propose that Poly-SNO-HSA does not cause kidney or liver damage and suggest that Poly-SNO-HSA may not interfere with cell cycle in nonmalignant cells.

## 5. Poly-SNO-HSA Can Overcome MDR

Cancer treatment remains one of the most important clinical challenges. One difficulty in treating various cancers is the development of multidrug resistance (MDR) by cancer cells. Various approaches have been tested to overcome MDR such as using agents that inhibit P-glycoprotein (P-gp) directly or indirectly through altering the cell membrane and using targeted drug delivery. Most of the approaches have shown some success in small animal models, but its clinical application has been limited. However, NO is one of the compounds that have shown promises in treating cancer.

**Figure 4:**
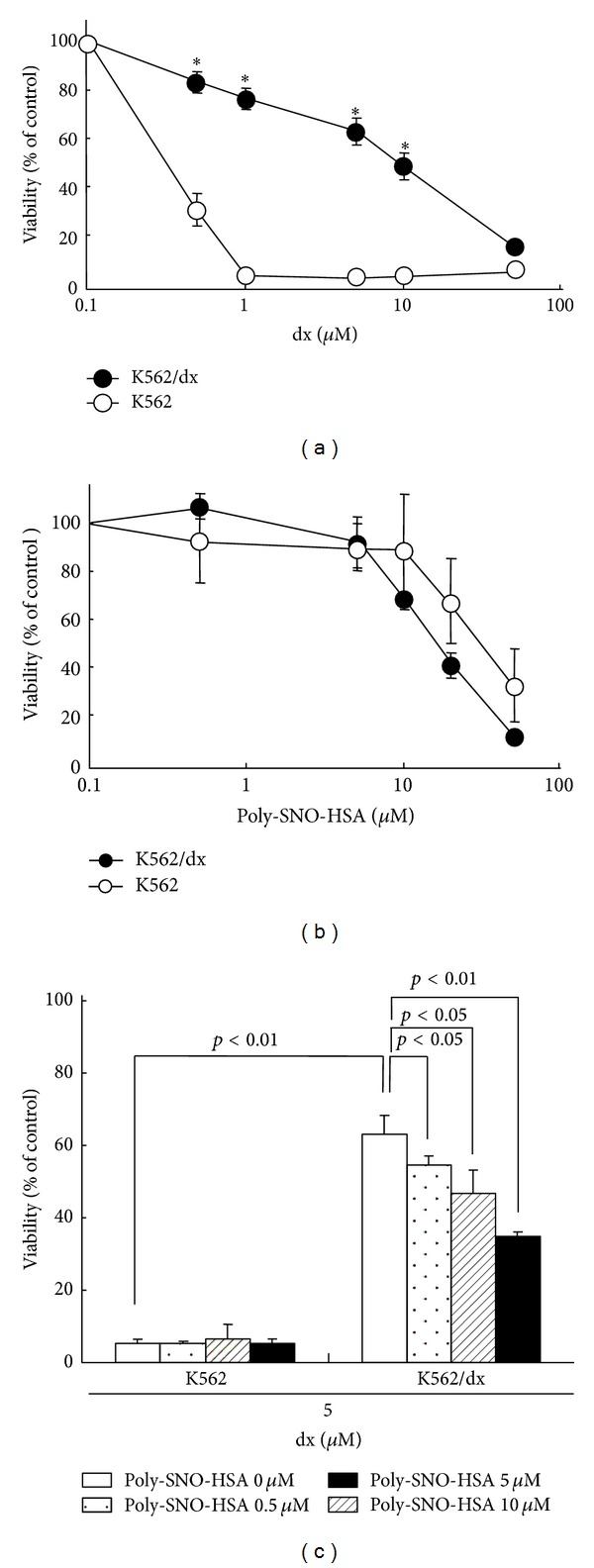
Synergistic effects of doxorubicin (dx) and Poly-SNO-HSA on the viability of K562 parental cells and doxorubicin-resistant K562 (K562/dx) cells. K562 cells (open circles) and K562/dx cells (closed circles) were treated for 24 h with various concentrations of dx (a) or Poly-SNO-HSA (b). In panel (c), K562 cells and K562/dx cells were treated with dx (5 *μ*M) for 24 h following incubation with various concentrations of Poly-SNO-HSA for 2  h. Data are expressed as means ± SD (*n* = 3). *P < 0.01, compared with K562 cells [29, 30].

In addition to its apoptosis effects, the antitumor effect of Poly-SNO-HSA could be due to its ability to overcome MDR. Therefore, we have performed *in vitro* and *in vivo* experiments to shed some light on this effect. For example, preliminary experiments showed, as expected, that doxorubicin- (dx-)resistant K562 (K562/dx) cells had significantly better survival as compared with K562 parental cells while treated with dx alone for 24 h (Figure 4(a)). To determine the effect of Poly-SNO-HSA on resistant tumor cell growth, the viability of K562 and K562/dx cells was examined after incubation with various concentrations of Poly-SNO-HSA. We found that Poly-SNO-HSA inhibited growth of both the K562 parental cells and the doxorubicin resistant K562/dx cells, in a similar way and in a dose-dependent manner (Figure 4(b)). Some of our earlier studies had shown that Poly-SNO-HSA also induces cell death in C26 cells and in LY-80 cells [29, 30]. In a following study, we first evaluated the transfer of NO from Poly-SNO-HSA into K562 cells and K562/dx cells by the DAF-FM DA fluorescence assay. It was seen that treatment of the cells with Poly-SNO-HSA resulted in a dose- and time-dependent uptake of NO the amount of which reached saturation in approximately 2 h. Therefore, in the following experiments different concentrations of Poly-SNO-HSA (0.5–10 *μ*M) were added to the cells 2 h before treatment with 5 *μ*M dx. The results showed a significant attenuation in the resistance to dx in the K562/dx cells as the concentration of Poly-SNO-HSA increased (Figure 4(c)). The inhibitory effects of Poly-SNO-HSA and dx on the growth of K562/dx cells were evaluated for synergistic action by using isobologram analysis. These results propose that Poly-SNO-HSA has the following two antitumor effects: it inhibits cell growth and attenuates the resistance to dx.

**Figure 5:**
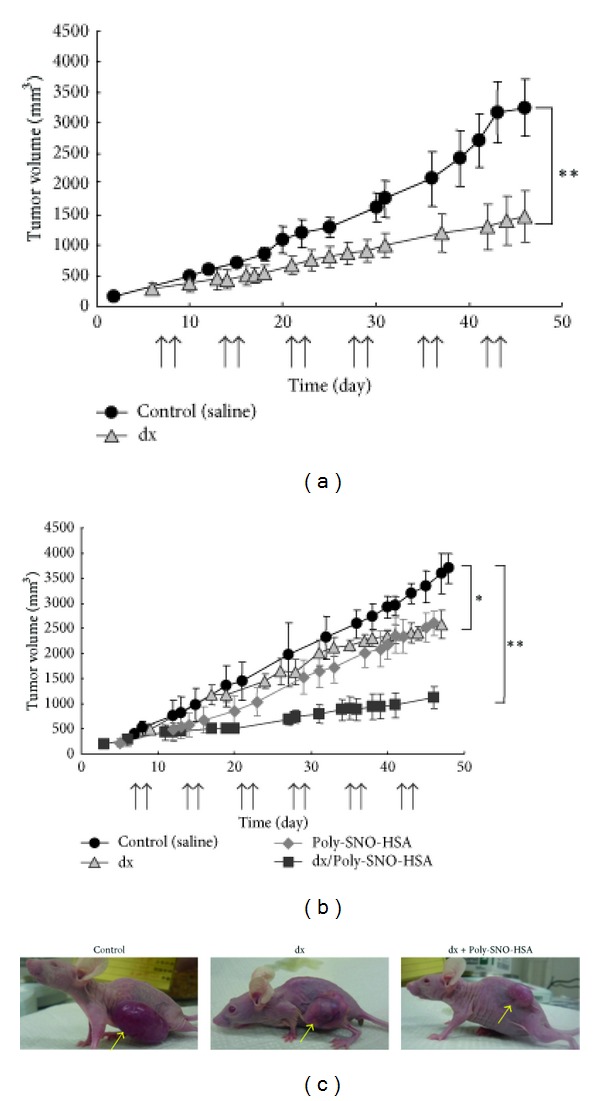
Effects of dx and Poly-SNO-HSA on tumor growth *in vivo*. K562 tumor-bearing mice (a) and K562/dx tumor-bearing mice (b) were given injections of saline (5 mL/kg), dx (4 mg/kg), Poly-SNO-HSA (10 *μ*mol/5 mL/kg), or dx combined with Poly-SNO-HSA biweekly as noted (↑). Results are means ± SD; *n* = 3-4 animals per experimental group. (c) Results are shown for one representative experiment of K562/dx tumor-bearing mice. *P < 0.05, **P < 0.01, compared with control (saline) [33].

To investigate the effect of Poly-SNO-HSA on chemoresistance *in vivo*, we prepared K562 and K562/dx tumor-bearing mice by injection of 2 × 10^7^ K562 or K562/dx cells into the left hind flank of female BALB/cAJcl-nu/nu mice. After the tumors had grown to 150–200 mm^3^, the mice received either intraperitoneal or intravenous injections of either saline, dx, and/or Poly-SNO-HSA biweekly. Treatment with dx decreased significantly the time-dependent tumor growth in K562 tumor-bearing mice (Figure 5(a)). By contrast, in K562/dx mice dx did not affect tumor growth significantly (Figure 5(b)). A slightly more pronounced, but significant, effect was observed, when the K562/dx mice were treated with Poly-SNO-HSA. However, combining dx and Poly-SNO-HSA decreased tumor volume to one-third as compared with treatment with them alone (Figure 5(c)). This observation proposes that Poly-SNO-HSA overcomes dx resistance *in vivo*. In the two types of mice, we also measured the tumor amounts of dx. The results showed that Poly-SNO-HSA increased several-fold the dx concentration in the tumor tissue of the K562/dx mice, suggesting that the antitumor effect of Poly-SNO-HSA was enhanced, at least in part, by increasing the local concentration of dx.

We have also examined the mechanism of Poly-SNO-HSA-induced chemosensitivity. NO shows multiple physiological actions via the cGMP-dependent way which involves activation of a soluble guanylate cyclase resulting in production of cGMP and activation of a protein kinase (PKG) [45, 46]. To determine whether the effect of Poly-SNO-HSA on resistant tumor cells was mediated by cGMP signaling, we used the soluble guanylate cyclase inhibitor 1-H-(1,2,4)oxadiazolo-(4,3-a)quinoxalin-1-one (ODQ) and the nonhydrolyzable cGMP analogue 8-Br-cGMP. ODQ in K562/dx cells suppressed the effect of Poly-SNO-HSA on reverting the resistance to dx. In contrast, the resistance to dx in K562/dx was significantly diminished following administration of 8-Br-cGMP. These data strongly indicate that Poly-SNO-HSA reverts dx resistance partly via the cGMP signaling pathway. Thus, activation of the NO-cGMP-dependent signaling pathway by NO donors or cGMP analogs could represent a novel approach to cancer therapy including chemoresistance.

Another mechanism of Poly-SNO-HSA-induced chemosensitivity could be intracellular accumulation of dx. Therefore, that potential effect was examined by flow cytometry [33]. We found that, after 1 h incubation with dx, the fluorescence intensity of dx in K562/dx cells was about 60% of that in K562 cells. Furthermore, in K562/dx cells, pretreatment for 2 h with 0.5–10 *μ*M Poly-SNO-HSA before dx addition significantly enhanced the dx accumulation.

In order to explain the modulating effects of Poly-SNO-HSA on dx in K562/dx cells, we also analyzed the expression of P-gp by Western blotting [33]. The P-gp protein was strongly expressed in K562/dx cells, while it was not clearly detected in K562 cells. The overexpression of P-gp in K562/dx was decreased by Poly-SNO-HSA pretreatment. These results indicate that Poly-SNO-HSA enhances intracellular accumulation of dx by downregulating P-gp.

The hypoxia-inducible factor-1*α* (HIF-1*α*) is activated in hypoxia solid tumor, and it is involved in regulating the transcription of the ATP-binding cassette transporters family including P-gp genes [47, 48]. To check for the involvement of HIF-1*α* in the above described effects of Poly-SNO-HSA, K562 cells were cultured for 24 h under hypoxic conditions [33]. Western blotting analysis showed clear HIF-1*α* expression in K562 cell lysates incubated to hypoxia. The high expression of HIF-1*α* was significantly suppressed in the presence of Poly-SNO-HSA. This finding is in full accordance with that observed for P-gp expression.

Similar results were obtained by immunostaining of P-gp and HIF-1*α* in K562 and K562/dx tumor-bearing mice [29].

**Figure 6:**
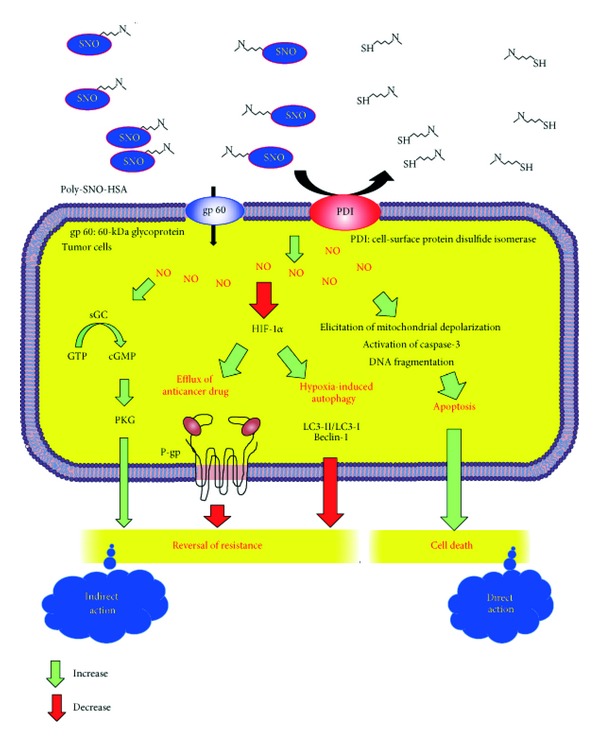
Mechanisms of Poly-SNO-HSA as a safe and strong multiple antitumor agent. Fast and pronounced transfer of NO from Poly-SNO-HSA into the cell mainly takes place via cell-surface protein disulfide isomerase (PDI) and to a minor extent via 60 kDa glycoprotein (gp60). Within the cell, a high concentration of NO induces apoptosis by the mechanisms mentioned, and perhaps by other means not yet identified. NO also reverts dx resistance partly by activating a cGMP dependent pathway. Finally, NO reverts drug resistance by decreasing the efflux of the drug. The latter effect is brought about by decreasing the expression of hypoxia-inducible factor-1*α* (HIF-1*α*) and P-glycoprotein (P-gp). In addition, Poly-SNO-HSA inhibits hypoxia-induced autophagy via downregulation of the cell signaling factors LC3-II/LC3-I and Beclin-1. The green and red arrows represent increasing and decreasing effects, respectively.

Finally, we checked the effects of normoxia and hypoxia conditions on the *in vitro* resistance of K562 cells to dx [33]. The data showed that hypoxia condition significantly induced resistance to dx (0.5–10 *μ*M). Intriguingly, Poly-SNO-HSA could inhibit growth of K562 cells under hypoxia condition in a dose-dependent manner, indicating that Poly-SNO-HSA inhibits the expression of HIF-1*α*, which is a major factor for the resistance to dx. This is the case for both hypoxia condition and for the K562/dx cells (Figure 6).

Our findings suggest that Poly-SNO-HSA can be developed as a safe and strong, multifunctional antitumor agent. This is especially true if the Poly-SNO-HSA can be formulated into suitable carriers targeted to tumors. One of the limitations of the current nanovehicles for targeted drug delivery is accumulation of the vehicles in nontarget organs, causing serious side effects. Since NO is rather benign unless the concentration is too high, it presents a unique opportunity to achieve treating tumors without significant side effects. The current approach of nanotechnology-based drug delivery to tumors can benefit significantly through understanding of the underlying mechanisms of drug actions, for example, therapeutic mechanisms of NO [33, 49].

## 6. Future Direction of Poly-SNO-HSA

The hypothesis that tumor progression can be curbed by antiangiogenic agents targeting abnormal tumor vessels is supported by preclinical evidence and clinical trials [50]. However, these initial successes were tempered by the failure of angiogenesis inhibitors to produce enduring clinical responses. For example, in clinical trials of the vascular endothelial growth factor- (VEGF-) neutralizing antibody bevacizumab in glioblastoma, 40% to 60% of the tumors progressed after initially successful treatment [51], consistent with the development of resistance to antiangiogenic therapy, a state exhibiting a poor prognosis and poor response to available treatments [52]. The molecular basis of acquired resistance to antiangiogenic treatments causing this lack of sustained responses remains unclear. However, Hu et al. [53] have demonstrated that the devascularization caused by antiangiogenic therapy increases tumor hypoxia and that this hypoxia mediates resistance to antiangiogenic therapy. Furthermore, recent reports suggest that hypoxia activates autophagy, a lysosomal degradation pathway which may promote tumor cell survival [54, 55]. In fact, the devascularization caused by antiangiogenic therapy increases tumor hypoxia, and this hypoxia mediates resistance to antiangiogenic therapy [53]. The mechanisms by which hypoxia induces autophagy need clarification, but the finding that BNIP3, a HIF-1*α* downstream target gene, is essential to hypoxia-induced autophagy suggests one possible mechanism [56, 57]. Therefore, autophagy inhibitors may help prevent resistance to antiangiogenic therapy. In our studies, we found that Poly-SNO-HSA possesses very strong HIF-1*α* inhibition in tumor cells *in vivo*. We hope that future studies will clarify whether Poly-SNO-HSA also can be used as a specific and potent autophagy inhibitor.

## References

[1] Moncada S, Palmer RMJ, Higgs EA (1991). Nitric oxide: physiology, pathophysiology, and pharmacology. *Pharmacological Reviews*.

[2] Mizutani T, Layon AJ (1996). Clinical applications of nitric oxide. *Chest*.

[3] Stamler JS, Singel DJ, Loscalzo J (1992). Biochemistry of nitric oxide and its redox-activated forms. *Science*.

[4] Nathan C (1992). Nitric oxide as a secretory product of mammalian cells. *FASEB Journal*.

[5] Lancaster JR (1994). Simulation of the diffusion and reaction of endogenously produced nitric oxide. *Proceedings of the National Academy of Sciences of the United States of America*.

[6] Gaston B, Reilly J, Drazen JM (1993). Endogenous nitrogen oxides and bronchodilator S-nitrosothiols in human airways. *Proceedings of the National Academy of Sciences of the United States of America*.

[7] Clancy RM, Levartovsky D, Leszczynska-Piziak J, Yegudin J, Abramson SB (1994). Nitric oxide reacts with intracellular glutathione and activates the hexose monophosphate shunt in human neutrophils: evidence for S- nitrosoglutathione as a bioactive intermediary. *Proceedings of the National Academy of Sciences of the United States of America*.

[8] Furchgott RF, Zawadzki JV (1980). The obligatory role of endothelial cells in the relaxation of arterial smooth muscle by acetylcholine. *Nature*.

[9] Palmer RMJ, Ferrige AG, Moncada S (1987). Nitric oxide release accounts for the biological activity of endothelium-derived relaxing factor. *Nature*.

[10] Radomski MW, Palmer RM, Moncada S (1987). The anti-aggregating properties of vascular endothelium: interactions between prostacyclin and nitric oxide. *British Journal of Pharmacology*.

[11] Dawson TM, Snyder SH (1994). Gases as biological messengers: nitric oxide and carbon monoxide in the brain. *Journal of Neuroscience*.

[12] Ignarro LJ, Napoli C, Loscalzo J (2002). Nitric oxide donors and cardiovascular agents modulating the bioactivity of nitric oxide: an overview. *Circulation Research*.

[13] Fung H-L (2004). Biochemical mechanism of nitroglycerin action and tolerance: is this old mystery solved?. *Annual Review of Pharmacology and Toxicology*.

[14] Ishima Y, Yoshida F, Kragh-Hansen U (2011). Cellular uptake mechanisms and responses to NO transferred from mono-and poly-S-nitrosated human serum albumin. *Free Radical Research*.

[15] Hibbs JB, Taintor RR, Vavrin Z, Rachlin EM (1988). Nitric oxide: a cytotoxic activated macrophage effector molecule. *Biochemical and Biophysical Research Communications*.

[16] Beckman JS, Crow JP (1993). Pathological implications of nitric oxide, superoxide and peroxynitrite formation. *Biochemical Society Transactions*.

[17] Isobe M, Katsuramaki T, Hirata K, Kimura H, Nagayama M, Matsuno T (1999). Beneficial effects of inducible nitric oxide synthase inhibitor on reperfusion injury in the pig liver. *Transplantation*.

[18] Beckman JS, Beckman TW, Chen J, Marshall PA, Freeman BA (1990). Apparent hydroxyl radical production by peroxynitrite: implications for endothelial injury from nitric oxide and superoxide. *Proceedings of the National Academy of Sciences of the United States of America*.

[19] Virág L, Szabó E, Gergely P, Szabó C (2003). Peroxynitrite-induced cytotoxicity: mechanism and opportunities for intervention. *Toxicology Letters*.

[20] Szabó C (2007). Hydrogen sulphide and its therapeutic potential. *Nature Reviews Drug Discovery*.

[21] Salvemini D, Jensen MP, Riley DP, Misko TP (1998). Therapeutic manipulations of peroxynitrite. *Drug News and Perspectives*.

[22] Friederich JA, Butterworth JF (1995). Sodium nitroprusside: twenty years and counting. *Anesthesia and Analgesia*.

[23] Robin ED, McCauley R (1992). Nitroprusside-related cyanide poisoning; time (long past due) for urgent, effective interventions. *Chest*.

[24] Saavedra JE, Billiar TR, Williams DL, Kim Y-M, Watkins SC, Keefer LK (1997). Targeting nitric oxide (NO) delivery in vivo. Design of a liver- selective NO donor prodrug that blocks tumor necrosis factor-*α*-induced apoptosis and toxicity in the liver. *Journal of Medicinal Chemistry*.

[25] Ricciardi R, Schaffer BK, Kim RD (2001). Protective effects of ischemic preconditioning on the cold-preserved liver are tyrosine kinase dependent. *Transplantation*.

[26] Liu J, Li C, Waalkes MP (2003). The nitric oxide donor, V-PYRRO/NO, protects against acetaminophen-induced hepatotoxicity in mice. *Hepatology*.

[27] Fiorucci S, Antonelli E, Tocchetti P, Morelli A (2004). Treatment of portal hypertension with NCX-1000, a liver-specific NO donor. A review of its current status. *Cardiovascular Drug Reviews*.

[28] Katsumi H, Nishikawa M, Hashida M (2007). Development of nitric oxide donors for the treatment of cardiovascular diseases. *Cardiovascular and Hematological Agents in Medicinal Chemistry*.

[29] Katayama N, Nakajou K, Komori H (2008). Design and evaluation of S-nitrosylated human serum albumin as a novel anticancer drug. *Journal of Pharmacology and Experimental Therapeutics*.

[30] Katayama N, Nakajou K, Ishima Y (2010). Nitrosylated human serum albumin (SNO-HSA) induces apoptosis in tumor cells. *Nitric Oxide*.

[31] Stamler JS, Jaraki O, Osborne J (1992). Nitric oxide circulates in mammalian plasma primarily as an S-nitroso adduct of serum albumin. *Proceedings of the National Academy of Sciences of the United States of America*.

[32] Marley R, Feelisch M, Holt S, Moore K (2000). A chemiluminescense-based assay for S-nitrosoalbumin and other plasma S-nitrosothiols. *Free Radical Research*.

[33] Ishima Y, Hara M, Kragh-Hansen U (2012). Elucidation of the therapeutic enhancer mechanism of poly-S-nitrosated human serum albumin against multidrug-resistant tumor in animal models. *Journal of Controlled Release*.

[34] Marks DS, Vita JA, Folts JD, Keaney JF, Welch GN, Loscalzo J (1995). Inhibition of neointimal proliferation in rabbits after vascular injury by a single treatment with a protein adduct of nitric oxide. *Journal of Clinical Investigation*.

[35] Ewing JF, Young DV, Janero DR, Garvey DS, Grinnell TA (1997). Nitrosylated bovine serum albumin derivatives as pharmacologically active nitric oxide congeners. *Journal of Pharmacology and Experimental Therapeutics*.

[36] Baek SH, Hrabie JA, Keefer LK (2002). Augmentation of intrapericardial nitric oxide level by a prolonged-release nitric oxide donor reduces luminal narrowing after porcine coronary angioplasty. *Circulation*.

[37] Katsumi H, Nishikawa M, Yamashita F, Hashida M (2005). Development of polyethylene glycol-conjugated poly-S-nitrosated serum albumin, a novel S-nitrosothiol for prolonged delivery of nitric oxide in the blood circulation in vivo. *Journal of Pharmacology and Experimental Therapeutics*.

[38] Hirst D, Robson T (2010). Nitric oxide in cancer therapeutics: interaction with cytotoxic chemotherapy. *Current Pharmaceutical Design*.

[39] Qin Y, Dey A, Purayil HT, Daaka Y (2013). Maintenance of androgen receptor inactivation by S-nitrosylation. *Cancer Research*.

[40] Kielbik M, Klink M, Brzezinska M, Szulc I, Sulowska Z (2013). Nitric oxide donors: spermine/NO and diethylenetriamine/NO induce ovarian cancer cell death and affect STAT3 and AKT signaling proteins. *Nitric Oxide*.

[41] Williams JL, Borgo S, Hasan I, Castillo E, Traganos F, Rigas B (2001). Nitric oxide-releasing nonsteroidal anti-inflammatory drugs (NSAIDs) alter the kinetics of human colon cancer cell lines more effectively than traditional NSAIDs: implications for colon cancer chemoprevention. *Cancer Research*.

[42] Gao J, Liu X, Rigas B (2005). Nitric oxide-donating aspirin induces apoptosis in human colon cancer cells through induction of oxidative stress. *Proceedings of the National Academy of Sciences of the United States of America*.

[43] Kogias E, Osterberg N, Baumer B (2012). Growth-inhibitory and chemosensitizing effects of the glutathione-S- transferase-*π*-activated nitric oxide donor PABA/NO in malignant gliomas. *International Journal of Cancer*.

[44] Duan S, Cai S, Yang Q, Forrest ML (2012). Multi-arm polymeric nanocarrier as a nitric oxide delivery platform for chemotherapy of head and neck squamous cell carcinoma. *Biomaterials*.

[45] Nagai H, Yasuda H, Hatachi Y (2012). Nitric oxide (NO) enhances pemetrexed cytotoxicity via NO–cGMP signaling in lung adenocarcinoma cells in vitro and in vivo. *International Journal of Oncology*.

[46] Hanafy KA, Krumenacker JS, Murad F (2001). NO, nitrotyrosine, and cyclic GMP in signal transduction. *Medical Science Monitor*.

[47] Quintero M, Mackenzie N, Brennan PA (2004). Hypoxia-inducible factor 1 (HIF-1) in cancer. *European Journal of Surgical Oncology*.

[48] Seeber LM, Zweemer RP, Verheijen RH, van Diest PJ (2010). Hypoxia-inducible factor-1 as a therapeutic target in endometrial cancer management. *Obstetrics and Gynecology International*.

[49] Otagiri M, Chuang VT, Maruyama T, Kragh-Hansen U (2013). Human serum albumin: new insights on its structural dynamics. *Functional Impacts and Pharmaceutical Applications*.

[50] Jain RK (2008). Lessons from multidisciplinary translational trials on anti-angiogenic therapy of cancer. *Nature Reviews Cancer*.

[51] Vredenburgh JJ, Desjardins A, Herndon JE (2007). Phase II trial of bevacizumab and irinotecan in recurrent malignant glioma. *Clinical Cancer Research*.

[52] Clark AJ, Lamborn KR, Butowski NA (2012). Neurosurgical management and prognosis of patients with glioblastoma that progress during bevacizumab treatment. *Neurosurgery*.

[53] Hu Y-L, DeLay M, Jahangiri A (2012). Hypoxia-induced autophagy promotes tumor cell survival and adaptation to antiangiogenic treatment in glioblastoma. *Cancer Research*.

[54] Mazure NM, Pouysségur J (2010). Hypoxia-induced autophagy: cell death or cell survival?. *Current Opinion in Cell Biology*.

[55] Leone RD, Amaravadi RK (2013). Autophagy: a targetable linchpin of cancer cell metabolism. *Trends in Endocrinology & Metabolism *.

[56] Tracy K, Dibling BC, Spike BT, Knabb JR, Schumacker P, Macleod KF (2007). BNIP3 is an RB/E2F target gene required for hypoxia-induced autophagy. *Molecular and Cellular Biology*.

[57] Hu YL, Jahangiri A, Delay M, Aghi MK (2012). Tumor cell autophagy as an adaptive response mediating resistance to treatments such as antiangiogenic therapy. *Cancer Research*.

